# Supplementary material for: The causal role of circulating inflammatory markers in osteoporosis: a bidirectional Mendelian randomized study
Source: Front Immunol. 2024 Jul 18;15:1412298. doi: 10.3389/fimmu.2024.1412298 (PMC11291241; doi:10.3389/fimmu.2024.1412298)
Supplement: Supplementary file 1 [file DataSheet_1.zip › supplementary material/OSTEOPOROSIS-CIM/GCST90274797.tsv.gz_leave_one_out.pdf]

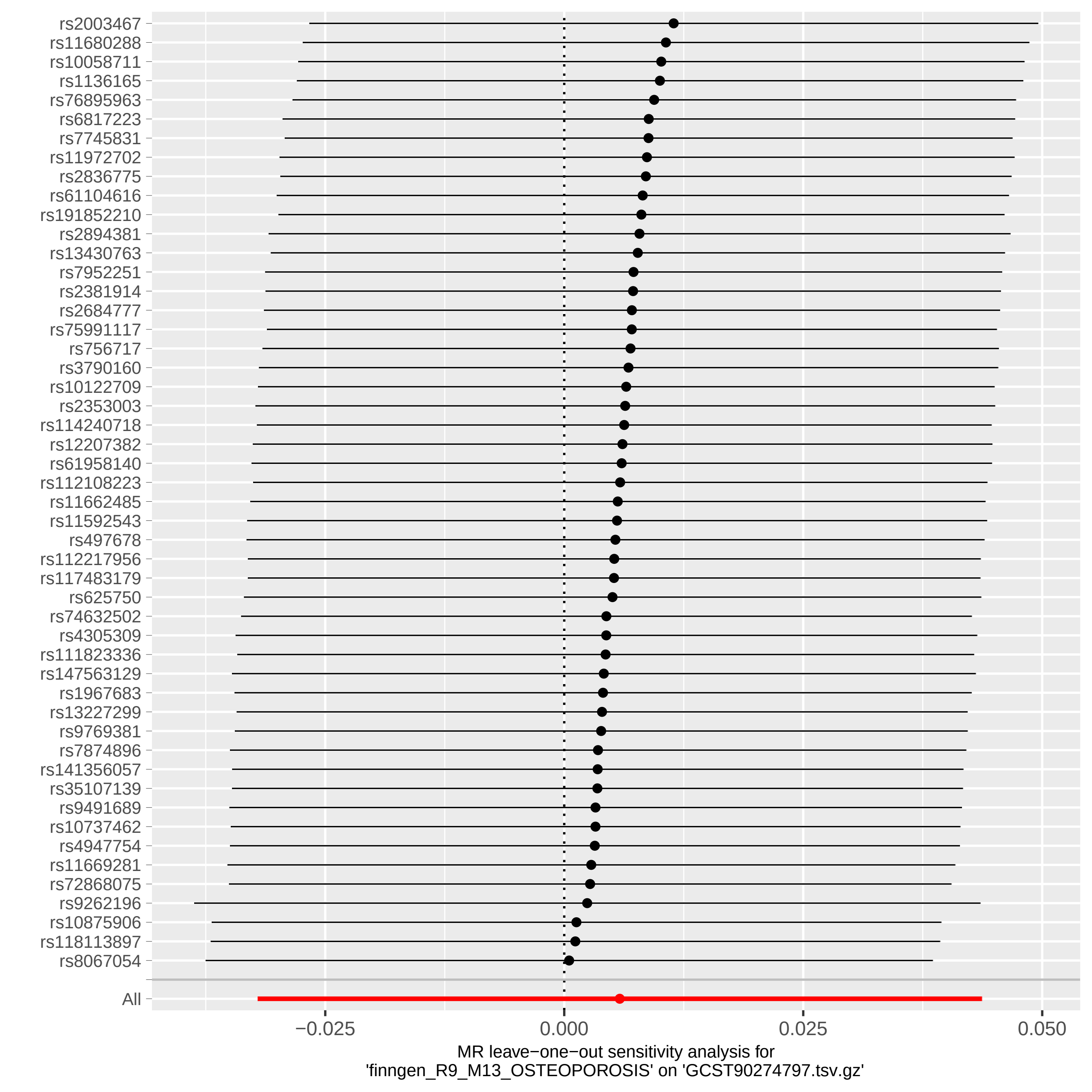
rs2003467
rs11680288
rs10058711
rs1136165
rs76895963
rs6817223
rs7745831
rs11972702
rs2836775
rs61104616
rs191852210
rs2894381
rs13430763
rs7952251
rs2381914
rs2684777
rs75991117
rs756717
rs3790160
rs10122709
rs2353003
rs114240718
rs12207382
rs61958140
rs112108223
rs11662485
rs11592543
rs497678
rs112217956
rs117483179
rs625750
rs74632502
rs4305309
rs111823336
rs147563129
rs1967683
rs13227299
rs9769381
rs7874896
rs141356057
rs35107139
rs9491689
rs10737462
rs4947754
rs11669281
rs72868075
rs9262196
rs10875906
rs118113897
rs8067054
All
−0.025
0.000
0.025
0.050
MR leave−one−out sensitivity analysis for
'finngen_R9_M13_OSTEOPOROSIS' on 'GCST90274797.tsv.gz'
